# The seroprevalence of brucellosis and molecular characterization of *Brucella* species circulating in the beef cattle herds in Albania

**DOI:** 10.1371/journal.pone.0229741

**Published:** 2020-03-05

**Authors:** Edi Fero, Arla Juma, Anita Koni, Jonida Boci, Toni Kirandjiski, Robert Connor, Gamal Wareth, Xhelil Koleci

**Affiliations:** 1 Department of Veterinary Public Health, Faculty of Veterinary Medicine, Agricultural University of Tirana, Tirana, Albania; 2 Animal Health Laboratory, Food Safety and Veterinary Institute, Tirana, Albania; 3 Animal Health Expert, ISUV, Tirana, Albania; 4 Institute of Bacterial Infections and Zoonoses, Friedrich-Loeffler-Institut, Jena, Germany; 5 Faculty of Veterinary Medicine, Benha University, Toukh, Qalyubia, Egypt; University of Lincoln, UNITED KINGDOM

## Abstract

**Background:**

Brucellosis is a ubiquitous zoonotic disease globally. It is endemic among bovines, sheep, and goats in Albania. The national control and eradication programs for brucellosis has been applied on sheep and goat farms as well as large dairy cattle farms, i.e., those with more than ten milking cows. The current study aims at estimating the herd and average individual animal prevalence of brucellosis in the national beef cattle herds, the missing information that was essential to propose the most appropriate control measures for this sub-population. Rose Bengal Test (RBT), Fluorescence Polarization Assay (FPA), and Enzyme-Linked Immunosorbent Assay (ELISA) were used as serological tests and classical bacteriology for isolation. Results were also used to investigate the difference in sensitivity between the assays used.

**Methodology:**

In total, 655 animals from 38 beef cattle herds from six southern districts of Albania were sampled. Sera were tested using RBT, FPA, and ELISA. Fifteen positive cows and a bull from eight high-prevalence positive herds were slaughtered, and particular tissue samples were collected for bacteriology.

**Results:**

The overall herd seroprevalence in the tested beef cattle population was 55%, while the overall average within-herd prevalence (including only positive herds) was 38.3%, 42.7%, and 45.6% determined by the RBT, ELISA, and FPA, respectively. FPA was used for the first time in the diagnosis of bovine brucellosis in Albania, and its sensitivity was higher than RBT and ELISA. Three *B*. *abortus* strains were identified, two from the supra-mammary lymph node of two cows and one from the epididymis of a seropositive bull.

**Conclusion:**

Brucellosis was highly prevalent in beef cattle in the southern part of Albania, and *B*. *abortus* was isolated from this subpopulation. To the best of our knowledge, this is the first statistically based survey of bovine brucellosis in beef herds in Albania. Using the FPA in parallel with other serological tests improved overall diagnostic sensitivity. Test and slaughter policy is not a rational approach for the control of brucellosis in beef cattle in Albania, and vaccination is only applicable, including strict control of the movement of animals.

## 1. Introduction

Brucellosis is a contagious bacterial disease that affects many domestic and wild animals, as well as humans. The World Health Organization (WHO) ranked it among the top seven neglected zoonoses [1]. In spite of farm animals playing the central role in transmission and maintenance of the infection, *Brucella* (*B*.) *abortus* has also been isolated from a dog and a cat in dairy cattle farms due to the ingestion of contaminated milk [2]. To date, there are 12 recognized species within the genus *Brucella*. The six classical species are *B*. *abortus*, *B*. *melitensis*, *B*. *suis*, *B*. *canis*, *B*. *ovis*, *B*. *neotomae* [3]. Two species of marine origin, *B*. *pinnipedialis* and *B*. *ceti*, were isolated from aquatic mammals [4]. *Brucella inopinata* [5] and *B*. *microti* [6] were isolated from humans and common voles, respectively. Recently, the isolation of *B*. *papionis* from baboons was described [7], and *B*. *vulpis* was isolated from mandibular lymph nodes of red foxes (*Vulpes vulpes*) [8]. *Brucella abortus*, *B*. *melitensis*, *B*. *suis*, and to some extent, *B*. *canis*, are responsible for the majority of infections in animals and humans. The disease causes substantial economic losses due to abortion in the last trimester, mastitis and reduced milk production in female animals, and orchitis and epididymitis in male animals. Infertility can occur in both male and female animals [9]. Brucellosis can be transmitted from animal to animal and from animals to humans by direct contact with infected animals or indirect contact with contaminated materials. The consumption of contaminated milk is still the main route of infection in humans [10]. The diagnosis of brucellosis is based mostly on the detection of specific antibodies in serum. However, no serological test has 100% diagnostic sensitivity and specificity [11]. In bovines, the Rose Bengal Test (RBT), Complement Fixation Test (CFT), and Enzyme-Linked Immunosorbent Assay (ELISA) are the most used serological assays. Recently, the Fluorescence Polarization Assay (FPA) has become available and is becoming more popular as a screening and confirmatory test. The test is based on the fluorescence polarization assay (FPA) technology to determine the presence of specific antibodies in serum, plasma, and milk samples [12]. In Albania, brucellosis is one of the most common zoonotic diseases and has both animal health and veterinary public health importance [13, 14]. Both *B*. *abortus* and *B*. *melitensis* affect the national cattle herd, small ruminants, and humans [15]. The control and eradication programs of brucellosis were directed at the reduction and elimination of the disease in small ruminants (sheep and goats), as well as in larger dairy cattle herds of more than ten animals [16]. In 2012, a national brucellosis control program in small ruminants started based on a mass vaccination campaign. It was repeated in 2013 and 2017, and from 2014 to 2016, the brucellosis control program was based on vaccination of replacement animals only. The first national program to control bovine brucellosis (BB) commenced in March 2016; it started with active surveillance in herds of more than 20 milking cows and, in 2018, was extended to include farms with more than ten milking cows. Passive surveillance started in smaller dairy herds and beef herds. Individual animals were tested in all positive herds, after which the standard control measures, including the slaughter of positive and in-contact animals, and cleaning and disinfection of the premises were implemented. Supplementary measures including enforcement of passive reporting and traceability were also enforced. The results of the implementation of the bovine brucellosis control program (BBCP) indicated an overall herd prevalence of less than 2% in dairy cattle [17, 18]. Investigation of bovine brucellosis in smaller herds and beef cattle within Albania has been neglected, and the scientific data are only available from sporadic, small-scale studies. Beef herds, which in Albania represent a small cattle subpopulation, are located mainly in the southern parts of the country and have not been included in active surveillance programs. It is generally suspected that these herds could be heavily infected by *Brucella* spp. but no information was available on a herd or within-herd prevalence rates, nor the causal agent. Thus, the study reported in this paper aimed to provide information on the herd and within-herd prevalence of brucellosis in beef cattle herds in Albania. In the event of the isolation of *Brucella* spp. from serologically positive animals, the identity of the specific *Brucella* pathogen which circulates within these herds were also investigated. Also, the study enabled an assessment of the practicability of the use of FPA for diagnosing brucellosis on a herd basis.

## 2. Materials and methods

### 2.1 Animal population and study design

This survey included only beef herds. The main characteristics of the production system in these herds are seasonal, employing natural insemination and using extensive grazing throughout most of the year. The geographical location of these herds is limited to the southern regions of Albania. While only a proportion of the total number of herds tested, the sample size was sufficient to estimate (±10%; d = 0.1) the proportion of infected herds with 95% (z = 1.960) confidence. It was assumed that 10% (p = 0.1; 1-p = 0.9) of herds were infected. Herds to be tested were selected from the sampling frame by simple random selection. No clustering effect was anticipated, and hence, no weighting included regarding the geographical location of the herds. To overcome cases where some of the farmers may have refused to submit their animals for sampling, and any other obstacles to the sampling of animals, an additional 50% (16 herds) were randomly selected. The following parameters were used to calculate the sample size to estimate within-herd prevalence (d = 0.1; z = 1.645; p = 0.5) and applied to each selected herd. Given the lack of individual ear tag numbers of animals within these herds, animals to be tested were selected on the spot. The inclusion criteria for animals to be tested were: females older than 12 months and all-male animals older than 12 months. Animals were randomly selected. The list of beef herds were drawn up from the national animal identification and registration database, in consultation with the regional veterinary office. The final list (sampling frame) had 517 herds with 10 to 360 animals per herd. A total of 38 beef cattle herds were selected using a simple random procedure, which was carried out in the six districts of the southern part of Albania [Delvina, Gjirokastër, Permet, Sarandë, Tepelenë, Vlora], where most of the country’s beef is produced.

### 2.2 Sera collection and serology

After obtaining owner consent, 655 eligible animals were sampled. A simple questionnaire was completed for each farm, and data on age, breed, animal movement, animal health status, and farm biosecurity were collected. Venous blood was collected from each animal into plain evacuated blood collection tubes from the caudal or jugular vein. Blood was allowed to clot, and the sera harvested after centrifugation of the clotted blood at 3,000 rpm for 5 minutes. Separated sera was stored in labeled screw-cap vials at -20°C. Each serum sample was subsequently tested in parallel utilizing RBT, ELISA, and FPA. The RBT was performed according to laboratory Standard Operating Procedures (SOP) based on the World Organization of Animal Health (OIE) manual [19]. Briefly, equal volumes (30 μL) of standardized *B*. *abortus* antigen and test serum were mixed thoroughly for 4 minutes. Any appearance of agglutination was recorded as a positive result. According to the degree of agglutination, positive samples classified as weakly positive (one (+)) to strongly positive (four plusses (++++)). The samples in which agglutination was not observed within 4 minutes were judged to be negative (−). The ELISA was performed using the IDEXX Brucellosis serum ab test (IDEXX Europe B.V., Hoofddorp, Netherland) according to the manufacturer's instructions. The criterion used for determining the status of animals based on ELISA was the S/P % value. The S/P % values <110 was considered negative, values between 110 and 120 considered inconclusive, while values greater than 120 were considered positive.

S/P % = $\frac{Sample\;A\;(450)-NC\bar{x}}{PC\bar{x}-NC\bar{x}}*100$(Sample A (450) = Sample Optical Density; $NC\bar{x}$ = mean value of negative control optical density: $PC\bar{x}$ = mean value of positive control optical density).

All samples were tested utilizing the FPA using *B*. *abortus* antibody test B1001 KIT (Ellie Headquarters Milwaukee, U.S.A United States). Sera was diluted in sample diluent at a 1:25 ratio. Briefly, the test procedure was performed in 10x75 mm borosilicate glass test tubes. FPA instrument glass tubes were used; to 20μl of each serum, samples, or control serum was added to 1 ml diluted samples diluent. Negative controls were run in triplicate, while each positive control and serum sample tested once only. After mixing, the samples were incubated (3–30 minutes) at room temperature, and a first (blank) reading obtained using Sentry® Software 2.3.26.exe. 10μl of the tracer was added to each sample and control. After 2–5 minutes, a second reading is done, and millipolarisation (mP) units obtained. The results of the FPA tests were expressed as delta mP (ΔmP) values of the samples and calculated as the difference between the mP value of the samples and the average of the negative controls mP values. The data was analyzed in Microsoft Excel with the add-in Data Analysis tool Pak (Descriptive Statistics). The 2x2 contingency table was used to calculate serological test parameters.

The criteria for classifying a herd as positive was based on a positive RBT result: if a herd had one positive, this was confirmed by either the FPA or ELISA result. The criteria for classifying individual animals as brucellosis-positive was based on a positive result in either the ELISA or FPA or both.

### 2.3 Isolation and bacterial identification

**Fig 1:**
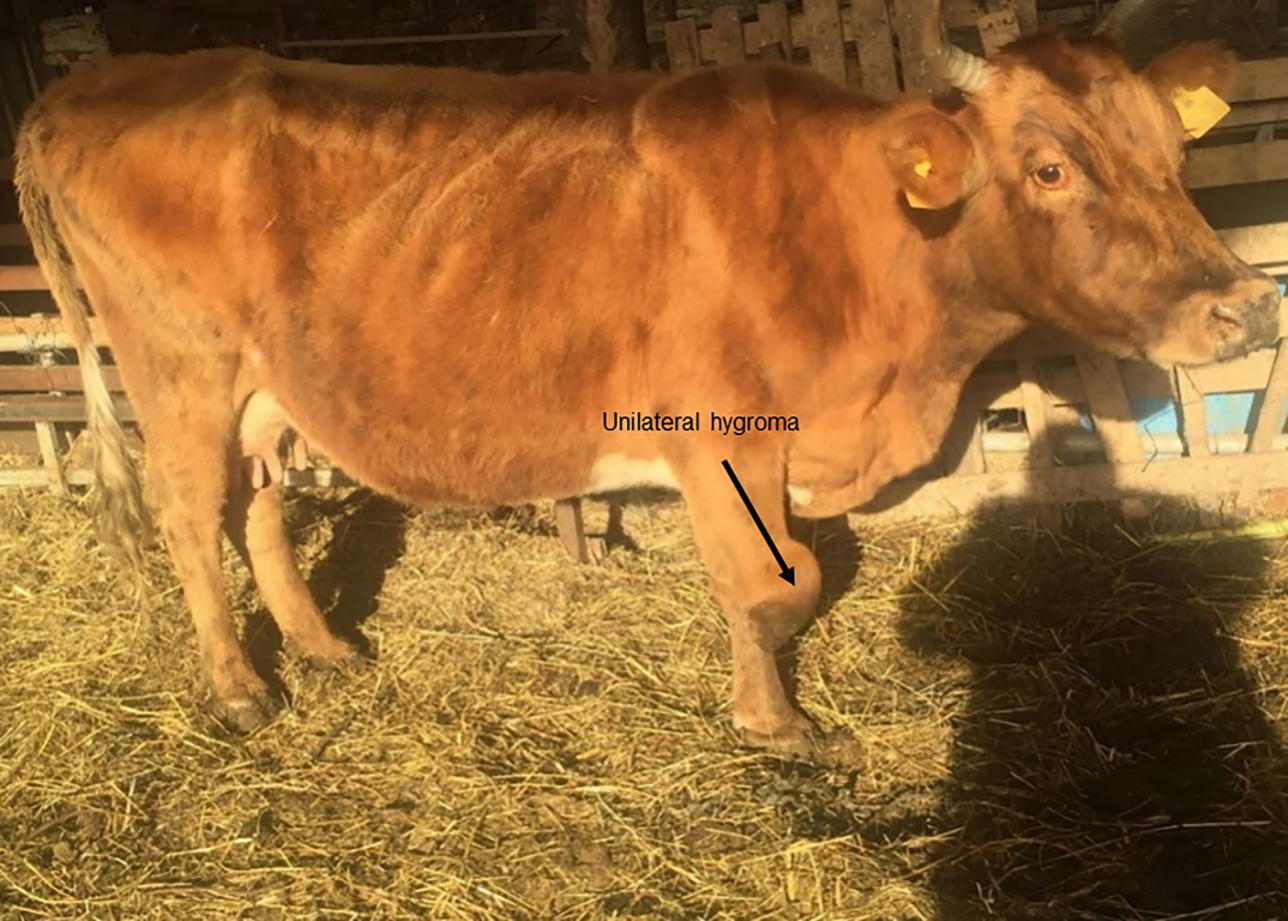
Unilateral hygroma on the knee joint (right carpal joint). This condition may be a consequence of bovine brucellosis. *Brucella abortus* was isolated from the supra-mammary lymph node of this cow.

In total, 15 seropositive cows and one bull from eight herds with high seroprevalence (more than 30%) were selected and slaughtered. Samples were collected for bacteriological isolation of *Brucella* spp. All selected animals were serologically positive, and some were from herds with abortion history, and one cow show enlargement of the knee joint **(Fig 1).** The animals were slaughtered in an approved slaughterhouse and tissue specimens taken from the supra-mammary lymph node (n = 15), spleen (n = 15), uterus (n = 8), and cotyledons (n = 7) of the seropositive cows. Also, spleen, testicular tissue, and epididymis were collected from a seropositive bull. All samples were collected and stored at -20°C before being transported under refrigeration to the bacteriology laboratory at the Faculty of Veterinary Medicine, Skopje, the Republic of Macedonia. *Brucella* identification and biotyping was carried out according to colony morphology, biochemical reaction including oxidase, catalase, and urease, CO_2_ requirement, H_2_S production, growth in the presence of thionin and fuchsine dyes, reaction with mono-specific anti-sera (A, M, R), agglutination with Trypaflavine and crystal-violet, and phage lyses as adopted by Alton et al. [20]. All suspected isolates were confirmed as *Brucella* spp. by molecular methods, *i*.*e*., by detection of IS711 using the *EU-RL* recommended method Real-Time PCR run according to SOP 596 for detection of IS711 gene [21]. Differentiation of *Brucella* species from vaccine Rev1, S19, and RB51 strains was done using *Bruce*-ladder as recommended by the OIE Manual of Diagnostic Tests and Vaccines for Terrestrial Animals 2017, Chapter 2.1.4 [19].

### 2.4 Ethics statement

All samples were collected after the agreement of the owners with the approval of the Chief Veterinary Officer of Albania. All experimental protocols were approved by the Animal Experiments and Ethics Committee of the Agricultural University of Tirana.

## 3. Results

### 3.1. Serological examination of the herds

In total, 38 herds were tested in the current study; 21 herds showed positive results in RBT, ELISA, and FPA, while 17 herds gave no positive reaction in any of the serological tests used. The highest number of positive herds (n = 8) were found in Sarandë district (Table 1) followed by Vlora (n = 5), Delvina (n = 4), and two herds in both Përmet and Gjirokastër. No positive herds were identified in the district of Tepelenë. The criterion used for determining the status of animals based on RBT results was the presence of visible agglutination. A positive reaction in the RBT was obtained with 147 out of 655 (22.4%) serum samples, while 508 (77.6%) were considered negative.

**Table 1:** Seroprevalence of bovine brucellosis in beef herds at the district level in southern Albania.

Districts	Tested herds	Positive herds	Overall herd prevalence	*SE	95% CI (lower)	95% CI (upper)	Average within-herd prevalence	Median within-herd prevalence
Delvina	4	4	100%	0.00	100%	100%	50%	51%
Gjirokastër	5	2	40%	0.22	-3%	83%	73%	73%
Permet	5	2	40%	0.22	-3%	83%	31%	31%
Sarandë	10	8	80%	0.13	55%	105%	52%	47%
Tepelenë	2	0	0%	0.00	0%	0%	0%	0%
Vlora	12	5	42%	0.14	14%	69%	40%	33%
**Total**	38	21	55%	0.08	40%	71%	28%	11%

*—SE: Standard error

**Fig 2:**
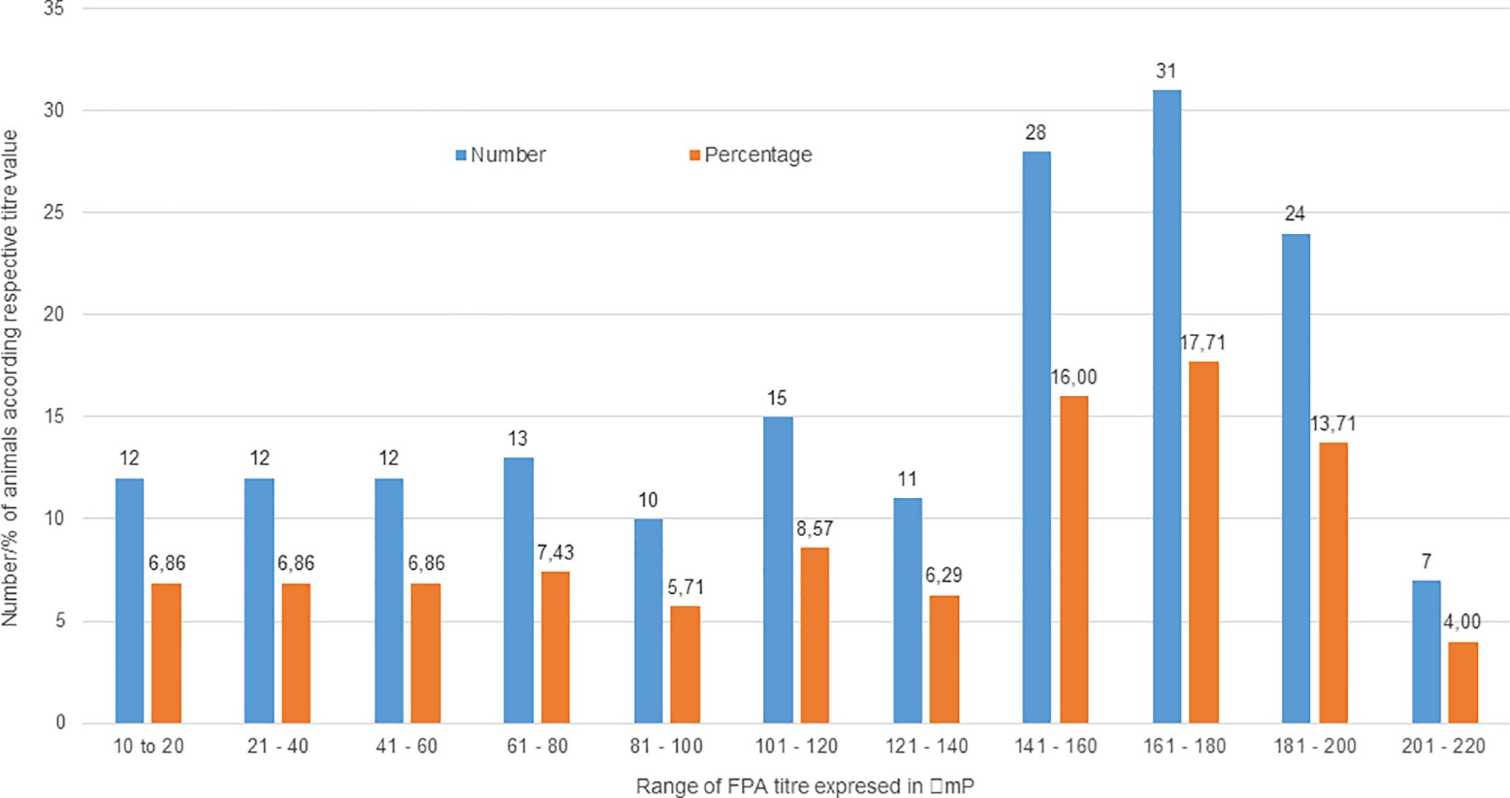
Histogram showing the distribution of FPA antibody titer to *Brucella abortus* in 175 positive animals. 116 (66.3%) animals showed a titer above 100 ΔmP. Also, the histogram shows a skewed distribution to the left-hand side, which indicates active infection, and these animals are likely to shed the bacteria into milk and other body fluids.

According to our ELISA results, 164 out of 655 animals (25%) gave S/P % value of ≥120 and were considered positive, while 484 (74%) animals gave S/P % value of ≤120 and were considered negative, while seven animals (1%) with values between 110 and 120 were deemed inconclusive. The criterion used for determining the status of animal was based on FPA titer expressed in ΔmP. The animals that produced a titer under 10 ΔmP were considered free of infection, while animals that showed a titer between 10 and 20 ΔmP were deemed to be doubtful (suspicious or suspect). Animals which produced a titer higher than 20 ΔmP were considered to be positive. The sera from all positive and suspect animals were re-tested in duplicate, and all samples in which at least one test produced a titer of more than 10 ΔmP were considered positive. 175 out of 655 animals (26.4%) were positive, while 480 in 655 (73.6%) animals were negative. A total of 116 (66.3%) animals showed a titer above 100 ΔmP, which indicates an active infection: these animals are likely to shed *Brucella* bacteria into milk or harbor them in body fluids. Also, the histogram shows a skewed distribution to the left-hand side **(Fig 2).** The number of positive animals and herd prevalences based on the results of RBT, FPA, and ELISA tests are shown in Table 2 according to the geographical distribution of sampling.

**Table 2:** Serological patterns found within the tested samples.

Tests results	Number of samples
RB+/ FPA- /ELISA-	5
RB-/ FPA+ /ELISA+	24
RB+/ FPA- /ELISA+	4
RB-/ FPA+ /ELISA-	9
RB+/ FPA+ /ELISA-	7
RB-/ FPA- /ELISA+	2

### 3.2 Results of comparison of serological tests

In 12 out of 21 infected herds (57.2%), a mismatch of the results between tests was found. Also on three other farms (D1, S8, and V7), the number of animals that tested positive in RBT was higher than in the confirmatory test (Table 3). The highest discrepancy was found in Delvina farms, followed by Sarandë, Gjirokastër, and Përmet. RBT produced at least five false-positive results (Table 2), while it did not detect 24 animals that were identified as positive by the confirmatory tests (ELISA and FPA). Besides, another four serological patterns were identified in the tested population. Sera from nine animals that were negative in both RBT and ELISA tests showed positive reactions in the FPA. Four animals were positive in the RBT and ELISA assay, but were negative in FPA. Seven animals were positive in RBT and FPA but were negative in ELISA, and two animals were negative in RBT and FPA but were positive in the ELISA test. Based on the above results, the calculation of the Kappa statistical coefficient to compare agreement between tests used would be informative [22]. The formula used to calculate the Kappa statistic is shown in Table 4.

**Table 3:** Results of RBT, FPA, and ELISA tests in the 38 beef herds according to the geographical distribution of sampling showing positive results in 21 herds.

District	Animals present on the farm	Tested animals per herd	RBT results		FPA results		ELISA results	
			No. of positive animals	Within herd prevalence	No. of positive animals	Within herd prevalence	No. of positive animals	Within herd prevalence
Delvinë-D1	35	19	12	63%	11	58%	11	58%
Delvinë-D2	17	12	6	50%	6	50%	6	50%
Delvinë-D3	20	14	4	29%	6	43%	6	43%
Delvinë-D4	55	28	9	32%	15	54%	15	54%
Gjirokastër -GJ1	20	14	0	0%	0	0%	0	0%
Gjirokastër-GJ2	16	13	0	0%	0	0%	0	0%
Gjirokastër-GJ3	20	15	0	0%	0	0%	0	0%
Gjirokastër-GJ4	110	32	19	59%	22	69%	21	66%
Gjirokastër-GJ5	13	9	6	67%	7	78%	5	56%
Permet-P1	26	18	2	11%	2	11%	2	11%
Permet-P2	21	14	0	0%	0	0%	0	0%
Permet-P3	17	14	0	0%	0	0%	0	0%
Permet -P4	21	16	6	38%	8	50%	6	38%
Permet-P5	15	11	0	0%	0	0%	0	0%
Sarandë-S1	180	35	15	43%	15	43%	16	46%
Sarandë-S2	21	16	10	63%	13	81%	11	69%
Sarandë-S3	178	37	13	35%	19	51%	17	46%
Sarandë-S4	13	9	2	22%	2	22%	1	11%
Sarandë-S5	97	30	0	0%	0	0%	0	0%
Sarandë-S6	220	1	1	*100%	1	100%	1	100%
Sarandë-S7	80	28	4	14%	9	32%	7	25%
Sarandë-S8	47	22	14	64%	12	55%	13	59%
Sarandë-S9	27	16	1	6%	3	19%	3	19%
Sarandë-S10	96	31	0	0%	0	0%	0	0%
Tepelenë-T1	26	19	0	0%	0	0%	0	0%
Tepelenë-T2	15	11	0	0%	0	0%	0	0%
Vlorë-V1	24	16	0	0%	0	0%	0	0%
Vlorë-V2	19	15	5	33%	5	33%	5	33%
Vlorë-V-3	22	15	1	7%	1	7%	1	7%
VlorëV-4	12	12	0	0%	0	0%	0	0%
Vlorë-V5	8	8	0	0%	0	0%	0	0%
Vlorë-V6	15	11	0	0%	0	0%	0	0%
Vlorë-V7	24	20	2	10%	1	5%	1	5%
Vlorë-V8	24	16	0	0%	0	0%	0	0%
Vlorë-V9	13	10	7	70%	7	70%	7	70%
Vlorë-V10	15	12	8	67%	10	83%	9	75%
Vlorë-V11	13	10	0	0%	0	0%	0	0%
Vlorë-V12	60	26	0	0%	0	0%	0	0%
**Total**	**1,655**	**655**	**147**	**22.4%**	**175**	**26.7%**	**164**	**25%**

*It was possible to sample only one animal from a beef herd with 250 animals.

**Table 4:** Kappa statistic between the RBT, ELISA, and FPA tests used in serodiagnosis of brucellosis in beef cattle herds in Albania.

Formulae	Combined tests		
	RBT and FPA	RBT and ELISA	ELISA and FPA
EP + = (a +b)/n x (a+c)/n	0.06	0.06	0.07
EP- = (c+d)/n x (b+d)n	0.57	0.58	0.55
*EP = EP*_*+*_ *+ EP-*	0.63	0.64	0.62
*MA = 1-EP*	0.37	0.36	0.38
*OP = (a+d)/n*	0.29	0.94	0.98
*OA = OP–EP*	0.92	0.30	0.36
***Kappa = OA/MA***	**0.79**	**0.83**	**0.94**

### 3.3 Calculation of the diagnostic parameters of RBT against FPA and ELISA tests

The sensitivity of the RBT test compared to FPA was 77.14% (95% CI 70.2–83.14%), the specificity was 97.50% (95% CI 95.67–98.70%), and the positive likelihood ratio (LR+) as a ratio between sensitivity/1-specificity was 30.86 (95% CI 17.55–54.26%). The negative likelihood ratio as the ratio between 1-sensitivity/specificity was 0.23 (95% CI 0.18–0.31). According to the data for RBT related to FPA, the individual disease prevalence was 26.72% (95% CI 23.36–30.28%), the positive predictive value was equal to 91.84% (86.48–95.19%), the negative predictive value was 92.13% (95% CI 89.91–93.89%), and test accuracy equal to 92.06% (95% CI 89.72–94.01%). The sensitivity of the RBT test compared to ELISA was 82.32% (95% CI 75.6–87.83%), the specificity was 97.56% (95% CI 95.77–98.73%), and the positive likelihood ratio (LR+) as the ratio between sensitivity/1-specificity was 33.68 (95% CI 19.18–59.16%), while the negative likelihood negative ratio as the ratio between 1-sensitivity/specificity was 0.18 (95% CI 0.13–0.25). According to the data for RBT related to ELISA, the disease prevalence was 25.4% (95% CI 21.76–28.54%), the positive predictive value equal to 91.84% (92.23–95.83%), the negative predictive value 94.29% (95% CI 92.23–95.83%), and the test accuracy was equal to 93.74% (95% CI 91.60–95.47%). The complete information and analysis of RBT, FPA and cELISA results shown in S1, S2 and S3 Tables.

### 3.4. Isolation and molecular identification

Three *Brucella* spp. strains have been isolated. Two were isolated from the supra-mammary lymph nodes of two cows originating from two different serologically positive herds, and one strain has been isolated from epididymis tissue of a bull originating from another third serologically positive herd. The serum from this bull produced a negative reaction for the RBT test, but positive for both the FPA and ELISA tests. The FPA titer results of the bull serum were low (13.2 ΔmP), and in the ELISA test, the sample was positive but did not have a strong positive result (142.7%). PCR identified all isolates as *B*. *abortus* (field strain), and no vaccine strains were detected (Table 5).

**Table 5:** Microbiological and serological results obtained from samples from the 16 slaughtered animals.

Sample ID	Serological test results			Microbiological test results (isolation)					
	RBT	FPA (ΔmP)	ELISA (S/P) value	Supra-mammary lymph node	Spleen	Uterus	Cotyledons	Epididymis	Testes
115	4	138.7	156.7	Negative	Negative	Negative	NI	NI	NI
116	4	178.0	146.9	Negative.	Negative	NI	Negative	NI	NI
129	4	174.5	170.5	**Positive**	Negative	NI	Negative	NI	NI
134	4	180.7	164.7	Negative	Negative	Negative	NI	NI	NI
196	Negative	191	Negative	Negative	Negative	Negative	NI	Ni	Ni
203	4	183.5	133.4	NI	Negative	NI	Negative	NI	NI
216	4	109.6	145.2	NI	Negative	Negative	NI	NI	NI
271	4	190.9	130.6	Negative	Negative	Negative	NI	NI	NI
272	Negative	119.1	142.4	Negative	Negative	Negative	NI	Ni	Ni
422	4	151.1	142.4	Negative	Negative	NI	Negative	NI	NI
440	2	167.2	145.1	Negative	Negative	NI	Negative	NI	NI
445	2	158.2	137.9	**Positive**	Negative	NI	Negative	NI	NI
479	4	151.1	142.9	Negative	Negative	Negative	NI	NI	NI
483	4	156.3	148.9	Negative	Negative	NI	Negative	NI	NI
503	4	181.5	134.9	Negative	Negative	Negative	NI	NI	NI
638	Negative	13.2	142.7	NI	Negative	NI	NI	**Positive**	Negative
**Number of included specimens**				**15**	**16**	**8**	**7**	**1**	**1**

NI: not included in the study.

## 4. Discussion

Brucellosis is a highly contagious bacterial zoonoses causing huge economic losses globally. The infection of a wide range of specific and nonspecific hosts extends plays a significant role in worldwide distribution of the disease [23]. An archaeological report assumed that brucellosis has been endemic in Albania since at least the Middle Ages. DNA sequencing revealed the presence of the *Brucella* IS6501 insertion element in skeletal remains from the ancient Albanian city of Butrint [24]. The disease is prevalent in humans and has been a significant infectious zoonotic disease for a long time [25]. However, the period from 2012 to 2016 showed a progressive decline in human cases, which coincided with veterinary interventions of mass vaccination [13]. The vaccination programs to control brucellosis in Albania have included only sheep and goat herds. Active surveillance was implemented in the larger dairy cattle herds (more than ten milking cows), while investigation and eradication of the disease in beef cattle farms and small herds of cattle were not sustainably applied. The current study is the first attempt to estimate the point prevalence of bovine brucellosis in beef cattle in Albania to propose the most appropriate control measures for this sub-population. The overall herd prevalence of bovine brucellosis in beef cattle, based on the results of RBT, FPA, and ELISA, was 55% (CI>0.95, 40–71%). It concluded that RBT, FPA, and ELISA are appropriate serological tests to identify infected herds and to estimate herd prevalence of brucellosis in beef cattle. Within-herd seroprevalences ranged from low (0%) to very high (100%): in nine herds, the individual animal prevalence was higher than 50%. FPA was the most sensitive test, followed by ELISA, and finally, RBT. FPA is recommended as an alternative and complementary test for the diagnosis of brucellosis either for screening during outbreaks or as a confirmatory test for individual animal diagnosis [26]. It was applied for the first time to diagnose bovine brucellosis in Albania in the current study. The analysis showed higher sensitivity and specificity compared to both the RBT and ELISA tests [27]. The test has been validated by many veterinary authorities [28]. It is standardized to be one of the main confirmatory tests for the diagnosis of brucellosis in cattle in most of the endemic countries of the Americas. It is approved in the European Union (EU) for testing cattle for trade between member states [29, 30]. Some large dairies use the FPA in programs to control and eradicate brucellosis as well as for testing for export and import purposes. Many other countries are now incorporating the *Brucella* FPA as either a confirmatory or a screening assay.

The highest herd seroprevalence was recorded in the Delvina district (100%); followed by the Sarandë district (80%), the Vlora district (42%), and the Gjirokastër and Përmet districts (40%), while in Tepelenë, no evidence of antibodies was detected in any tested animals (Table 1). These results are similar to those reported from surveys in other countries [31], but are higher than the results reported from another survey [32]. The traditional pastoral management system of beef cattle in Albania facilitates the spread of disease. The previous lack of a systematic brucellosis control program and the absence of effective animal movement controls significantly contribute to the spread of bovine brucellosis in this cattle subpopulation. These findings for beef cattle reflect the results of a survey of dairy cattle, where the highest seroprevalence was reported in Delvina district [33].

The serological test results need to be interpreted carefully, and an epidemiological analysis must be considered before any decision is reached on a final disease diagnosis. It is advisable to combine two or more serological tests. It is recommended to use the FPA to support a test and cull policy and the ELISA to verify the negative status. FPA can determine the titer of specific antibodies to *Brucella* spp. The distribution titers at the population level could be used to discriminate vaccinated from infected animals: in a vaccinated animal, the serological titers are lower than an infected cow. If the titer is skewed to the left, the herd can be assumed to be infected, and if the titer is skewed to the right, the herd can be expected to be vaccinated. In addition to the diagnosis of bovine brucellosis, FPA is validated for the determination of porcine brucellosis. It is recommended as a confirmatory test at the individual animal level for surveillance and to reduce the cross-reactivity with *Yersinia enterocolitica* O9 [26].

In this study, *Brucella* was isolated from the supramammary tissue of two seropositive cows and the epididymis of a seropositive bull. The results indicate that *B*. *abortus* was circulating in this subpopulation of beef cattle in the southern part of Albania. All isolates were identified as the *B*. *abortus* field strain. In beef production, milk is generally used for the suckling calf, which poses a risk of transmission of infection to newborn animals, as the vertical mode could transmit it. Also, low hygiene standards are predominant in these herds, hence licking of fetuses, ingestion of fetal membranes, contaminated feed, and water are common routes of infection transmission. Transmission by inhalation is also possible, and it is a more effective route since the infective dose is lower than for the digestive route [34]. The risk of transmission of brucellosis to humans from beef cattle is less than the risk from dairy cattle because milk from beef cows is destined for the calf rather than for human consumption. However, the farmers and farm workers may use the milk for their consumption. Muscle is not a preferred site for *Brucella* spp., however specific organs such as the spleen, liver, and lymph nodes are target tissues. During calving season, beef cattle are kept on pasture where, during calving, large quantities of bacteria are shed, which heavily contaminate the environment. The control of animal movement must be strictly enforced to avoid the introduction of *Brucella* infections from an infected beef herd to the infection-free beef /dairy herds. One cow in this study had a carpal hygroma, which is rare but typical for chronic brucellosis [35]. This pathology plays no direct role in the spread of disease in animals, though abattoir workers and individuals who conduct informal slaughter may become infected. *Brucella abortus* strain was isolated from the epididymis of a seven-year-old bull, where no visible inflammation or lesions were observed in the epididymal tissue. An infected bull poses a risk of transmission of infection by the venereal route.

Molecular methods are suitable for detecting the presence of DNA of *Brucella* spp., and it is more sensitive than the isolation method. Still, these methods cannot discriminate genetic material from live or dead bacteria. The biotyping method recommended by the World Organization for Animal Health (OIE) relies on serology and requires live bacteria to classify *Brucella* at the biovar level. However recently, whole genomic sequencing (WGS) typing tools have become available in several laboratories and can discriminate between *Brucella* strains and provide higher resolution genetic clustering as well as providing a useful tool in tracing back the geographic origin of infection from an unknown source [36]. Based on serological results, where the seroprevalence is found to be quite high, we suggest that vaccination with *Brucella abortus* S19, administered by the intra-conjunctival route would be the most rational policy for the control of bovine brucellosis in beef cattle. However, it must be accompanied by strict control of animal movement and other accompanying measures. The number of animals slaughtered in this study was relatively low compared to the 25 animals that it was planned to slaughter, there is a need for a system to be in a place that encourages the owner of beef herds to report abortion cases and perform microbiological tests. The target samples must be collected from slaughtered beef cattle based on risk and combine the results of at least two serological tests: the isolation procedure and the molecular analysis must be performed in parallel.

## 5. Conclusions

Bovine brucellosis is highly prevalent in beef cattle, both at the herd and within-herd level. *Brucella abortus* was isolated from seropositive animals and seemed to circulate in the beef cattle subpopulation. Given the very high prevalence in beef herds, the most efficient and affordable control approach is by mass vaccination. Under these conditions, the use of the intra-conjunctival S19 vaccine is most appropriate. Rigorous animal movement control is required to avoid the incompatibility of immunization with the test and slaughter approach.

## Supporting information

S1 Table Analyses of the RBT test results. (DOCX)

S2 Table Analyses of the FPA test results. (DOCX)

S3 Table Analyses of the c-ELISA test results. (DOCX)
