# Supplementary material for: The seroprevalence of brucellosis and molecular characterization of Brucella species circulating in the beef cattle herds in Albania
Source: PLoS One. 2020 Mar 5;15(3):e0229741. doi: 10.1371/journal.pone.0229741 (PMC7058276; doi:10.1371/journal.pone.0229741)
Supplement: S1 Table — (DOCX) [file pone.0229741.s001.docx]

**Supplementary table 1:** Analyses of the RBT test results

| **District** | **Animals present on the farm** | **Tested animals** | **Farm positive** | **Number of positive animals** | **Required min SS to estimate prevalence (90%CI; p=.5; d=.15)** | **SS achieved** | **Within-herd prevalence** | **SE** | **90% CI (lower)** | **90% CI (upper)** |
| --- | --- | --- | --- | --- | --- | --- | --- | --- | --- | --- |
| Delvinë | 35 | 19 | 1 | 12 | 16 | 3 | 63% | 0.11 | 45% | 81% |
| Delvinë | 17 | 12 | 1 | 6 | 11 | 1 | 50% | 0.14 | 27% | 73% |
| Delvinë | 20 | 14 | 1 | 4 | 12 | 2 | 29% | 0.13 | 7% | 50% |
| Delvinë | 55 | 28 | 1 | 9 | 19 | 9 | 32% | 0.09 | 17% | 47% |
| Gjirokastër | 20 | 14 | 0 | 0 | 12 | 2 | 0% | 0.00 | 0% | 0% |
| Gjirokastër | 16 | 13 | 0 | 0 | 10 | 3 | 0% | 0.00 | 0% | 0% |
| Gjirokastër | 20 | 15 | 0 | 0 | 12 | 3 | 0% | 0.00 | 0% | 0% |
| Gjirokastër | 110 | 32 | 1 | 19 | 24 | 8 | 59% | 0.08 | 46% | 72% |
| Gjirokastër | 13 | 9 | 1 | 6 | 9 | 0 | 67% | 0.14 | 44% | 90% |
| Permet | 26 | 18 | 1 | 2 | 14 | 4 | 11% | 0.07 | 0% | 23% |
| Permet | 21 | 14 | 0 | 0 | 12 | 2 | 0% | 0.00 | 0% | 0% |
| Permet | 17 | 14 | 0 | 0 | 11 | 3 | 0% | 0.00 | 0% | 0% |
| Permet | 21 | 16 | 1 | 6 | 12 | 4 | 38% | 0.13 | 16% | 59% |
| Permet | 15 | 11 | 0 | 0 | 10 | 1 | 0% | 0.00 | 0% | 0% |
| Sarandë | 180 | 35 | 1 | 15 | 26 | 9 | 43% | 0.08 | 30% | 56% |
| Sarandë | 21 | 16 | 1 | 10 | 12 | 4 | 63% | 0.10 | 46% | 79% |
| Sarandë | 178 | 37 | 1 | 13 | 26 | 11 | 35% | 0.08 | 22% | 48% |
| Sarandë | 13 | 9 | 1 | 2 | 9 | 0 | 22% | 0.14 | -1% | 45% |
| Sarandë | 97 | 30 | 0 | 0 | 23 | 7 | 0% | 0.00 | 0% | 0% |
| Sarandë | 220 | 1 | 1 | 1 | 26 | -25 | 100%^^[[1]](#footnote-1)^^ |  | 100% | 100% |
| Sarandë | 80 | 28 | 1 | 4 | 22 | 6 | 14% | 0.09 | 0% | 29% |
| Sarandë | 47 | 22 | 1 | 14 | 18 | 4 | 64% | 0.11 | 46% | 82% |
| Sarandë | 27 | 16 | 1 | 1 | 14 | 2 | 6% | 0.10 | -10% | 23% |
| Sarandë | 96 | 31 | 0 | 0 | 23 | 8 | 0% | 0.00 | 0% | 0% |
| Tepelenë | 26 | 19 | 0 | 0 | 14 | 5 | 0% | 0.00 | 0% | 0% |
| Tepelenë | 15 | 11 | 0 | 0 | 10 | 1 | 0% | 0.00 | 0% | 0% |
| Vlorë | 24 | 16 | 0 | 0 | 13 | 3 | 0% | 0.00 | 0% | 0% |
| Vlorë | 19 | 15 | 1 | 5 | 12 | 3 | 33% | 0.12 | 14% | 53% |
| Vlorë | 22 | 15 | 1 | 1 | 13 | 2 | 7% | 0.07 | -5% | 18% |
| Vlorë | 12 | 12 | 0 | 0 | 9 | 3 | 0% | 0.00 | 0% | 0% |
| Vlorë | 8 | 8 | 0 | 0 | 6 | 2 | 0% | 0.00 | 0% | 0% |
| Vlorë | 15 | 11 | 0 | 0 | 10 | 1 | 0% | 0.00 | 0% | 0% |
| Vlorë | 24 | 20 | 1 | 2 | 13 | 7 | 10% | 0.05 | 2% | 18% |
| Vlorë | 24 | 16 | 0 | 0 | 13 | 3 | 0% | 0.00 | 0% | 0% |
| Vlorë | 13 | 10 | 1 | 7 | 9 | 1 | 70% | 0.14 | 47% | 93% |
| Vlorë | 15 | 12 | 1 | 8 | 10 | 2 | 67% | 0.11 | 49% | 85% |
| Vlorë | 13 | 10 | 0 | 0 | 9 | 1 | 0% | 0.00 | 0% | 0% |
| Vlorë | 60 | 26 | 0 | 0 | 20 | 6 | 0% | 0.00 | 0% | 0% |
|  | **1,655** | **655** | **21** | **147** |  |  | **22.4%** |  |  |  |

1. From this beef herd only one cow was sampled and tested, because the circumstance did not allow animal handling directly on the pasture [↑](#footnote-ref-1)
