# Supplementary material for: The seroprevalence of brucellosis and molecular characterization of Brucella species circulating in the beef cattle herds in Albania
Source: PLoS One. 2020 Mar 5;15(3):e0229741. doi: 10.1371/journal.pone.0229741 (PMC7058276; doi:10.1371/journal.pone.0229741)
Supplement: S3 Table — (DOCX) [file pone.0229741.s003.docx]

**Supplementary table 3: Analyses of the ELISA test results**

| District | Animals present on the farm | Tested animals | Farm positive | Number of positive animals | Required min SS to estimate prevalence (90%CI; p=.5; d=.15) | SS achieved | Within-herd prevalence | SE | 90% CI (lower) | 90% CI (upper) |
| --- | --- | --- | --- | --- | --- | --- | --- | --- | --- | --- |
| Delvinë | 35 | 19 | 1 | 11 | 16 | 3 | 58% | 0.11 | 40% | 76% |
| Delvinë | 17 | 12 | 1 | 6 | 11 | 1 | 50% | 0.14 | 27% | 73% |
| Delvinë | 20 | 14 | 1 | 6 | 12 | 2 | 43% | 0.13 | 22% | 64% |
| Delvinë | 55 | 28 | 1 | 15 | 19 | 9 | 54% | 0.09 | 39% | 68% |
| Gjirokastër | 20 | 14 | 0 | 0 | 12 | 2 | 0% | 0.00 | 0% | 0% |
| Gjirokastër | 16 | 13 | 0 | 0 | 10 | 3 | 0% | 0.00 | 0% | 0% |
| Gjirokastër | 20 | 15 | 0 | 0 | 12 | 3 | 0% | 0.00 | 0% | 0% |
| Gjirokastër | 110 | 32 | 1 | 21 | 24 | 8 | 66% | 0.08 | 53% | 79% |
| Gjirokastër | 13 | 9 | 1 | 5 | 9 | 0 | 56% | 0.14 | 33% | 79% |
| Permet | 26 | 18 | 1 | 2 | 14 | 4 | 11% | 0.07 | 0% | 23% |
| Permet | 21 | 14 | 0 | 0 | 12 | 2 | 0% | 0.00 | 0% | 0% |
| Permet | 17 | 14 | 0 | 0 | 11 | 3 | 0% | 0.00 | 0% | 0% |
| Permet | 21 | 16 | 1 | 6 | 12 | 4 | 38% | 0.13 | 16% | 59% |
| Permet | 15 | 11 | 0 | 0 | 10 | 1 | 0% | 0.00 | 0% | 0% |
| Sarandë | 180 | 35 | 1 | 16 | 26 | 9 | 46% | 0.08 | 33% | 59% |
| Sarandë | 21 | 16 | 1 | 11 | 12 | 4 | 69% | 0.10 | 52% | 85% |
| Sarandë | 178 | 37 | 1 | 17 | 26 | 11 | 46% | 0.08 | 33% | 59% |
| Sarandë | 13 | 9 | 1 | 1 | 9 | 0 | 11% | 0.14 | -12% | 34% |
| Sarandë | 97 | 30 | 0 | 0 | 23 | 7 | 0% | 0.00 | 0% | 0% |
| Sarandë | 220 | 1 | 1 | 1 | 26 | -25 | 100% |  | 100% | 100% |
| Sarandë | 80 | 28 | 1 | 7 | 22 | 6 | 25% | 0.09 | 10% | 40% |
| Sarandë | 47 | 22 | 1 | 13 | 18 | 4 | 59% | 0.11 | 41% | 77% |
| Sarandë | 27 | 16 | 1 | 3 | 14 | 2 | 19% | 0.10 | 2% | 35% |
| Sarandë | 96 | 31 | 0 | 0 | 23 | 8 | 0% | 0.00 | 0% | 0% |
| Tepelenë | 26 | 19 | 0 | 0 | 14 | 5 | 0% | 0.00 | 0% | 0% |
| Tepelenë | 15 | 11 | 0 | 0 | 10 | 1 | 0% | 0.00 | 0% | 0% |
| Vlorë | 24 | 16 | 0 | 0 | 13 | 3 | 0% | 0.00 | 0% | 0% |
| Vlorë | 19 | 15 | 1 | 5 | 12 | 3 | 33% | 0.12 | 14% | 53% |
| Vlorë | 22 | 15 | 1 | 1 | 13 | 2 | 7% | 0.07 | -5% | 18% |
| Vlorë | 12 | 12 | 0 | 0 | 9 | 3 | 0% | 0.00 | 0% | 0% |
| Vlorë | 8 | 8 | 0 | 0 | 6 | 2 | 0% | 0.00 | 0% | 0% |
| Vlorë | 15 | 11 | 0 | 0 | 10 | 1 | 0% | 0.00 | 0% | 0% |
| Vlorë | 24 | 20 | 1 | 1 | 13 | 7 | 5% | 0.05 | -3% | 13% |
| Vlorë | 24 | 16 | 0 | 0 | 13 | 3 | 0% | 0.00 | 0% | 0% |
| Vlorë | 13 | 10 | 1 | 7 | 9 | 1 | 70% | 0.14 | 47% | 93% |
| Vlorë | 15 | 12 | 1 | 9 | 10 | 2 | 75% | 0.11 | 57% | 93% |
| Vlorë | 13 | 10 | 0 | 0 | 9 | 1 | 0% | 0.00 | 0% | 0% |
| Vlorë | 60 | 26 | 0 | 0 | 20 | 6 | 0% | 0.00 | 0% | 0% |
|  | **1,655** | **655** | **21** | **164** |  |  | **25%** |  |  |  |
